# Risk Mapping of African Swine Fever in Domestic Pigs and Wild Boars to Enhance Management and Surveillance in Asia

**DOI:** 10.1155/tbed/8850856

**Published:** 2025-11-21

**Authors:** Nijiho Kawaguchi, Cecilia Aguilar-Vega, Michihito Sasaki, Yasuko Orba, Hirofumi Sawa, José Manuel Sánchez-Vizcaíno, Norikazu Isoda, Jaime Bosch, Satoshi Ito

**Affiliations:** ^1^VISAVET Health Surveillance Center, Complutense University of Madrid, Madrid, Spain; ^2^Department of Animal Health, Faculty of Veterinary Medicine, Complutense University of Madrid, Madrid, Spain; ^3^Division of Molecular Pathobiology, International Institute for Zoonosis Control, Hokkaido University, Sapporo, Japan; ^4^Institute for Vaccine Research and Development, Hokkaido University, Sapporo, Japan; ^5^International Collaboration Unit, International Institute for Zoonosis Control, Hokkaido University, Sapporo, Japan; ^6^One Health Research Center, Hokkaido University, Sapporo, Japan; ^7^Global Virus Network, Baltimore, Maryland, USA; ^8^Laboratory of Microbiology, Faculty of Veterinary Medicine, Hokkaido University, Sapporo, Japan; ^9^South Kyushu Livestock Veterinary Medicine Center, Joint Faculty of Veterinary Medicine, Kagoshima University, Soo, Japan

**Keywords:** African swine fever, Asia, domestic pigs, epidemiology, GIS, risk assessment, *Sus scrofa*, underreporting, wild boar, wildlife management

## Abstract

African swine fever (ASF) is a highly lethal disease affecting domestic pigs and wild boars, caused by the ASF virus (ASFV), which has rapidly spread across Asia in recent years. In this region, most reported ASF cases involve domestic pigs, while cases in wild boars remain notably lower except in a few countries. However, factors such as the high population of wild boars, limited wildlife surveillance, and inadequate farm biosecurity suggest that the prevalence and transmission of ASFV between these hosts may be underestimated. Therefore, we used a simplified multicriteria approach (SMCA) to identify vulnerable areas (VAs) for ASFV infection and validated the resulting VA maps with chi-square tests using reported ASF cases. The spatial SMCA revealed that VAs for ASFV infection in domestic pigs are concentrated in eastern China, while high-risk zones for ASFV infection in wild boars span Russia, eastern China, and Southeast Asia. Sensitity analysis showed that the variables that most influenced the risk of ASFV infection in domestic pigs and wild boars were anthropogenic factors and distribution of wild boars, respectively. Additionally, we predicted areas with significant transmission potential between domestic pigs and wild boars. High-risk regions for interspecies transmission include eastern China, southwestern Korea, and southern Japan. This study offers a standardized method to assess ASFV infection risk across Asia by integrating environmental and anthropogenic factors rather than relying solely on reported outbreaks. The findings highlight potential high-risk regions, including those without detected outbreaks, to improve surveillance and early detection strategies.

## 1. Introduction

African swine fever (ASF), caused by the ASF virus (ASFV), is a contagious viral hemorrhagic disease that affects only suids and has a significant negative impact on the global pig industry. ASFV is a large (170–193 kbp), double-stranded DNA virus belonging to the *Asfarviridae* family [1]. Depending on the virulence of the virus, transmission route, and host immunity, ASF presents a range of clinical signs and pathological lesions, classified into four main forms: peracute (death within 1–4 days postinfection), acute (death within 3–15 days), subacute (death within 20 days), and chronic (low mortality with some survivors becoming lifelong carriers) [2]. Domestic pigs and Eurasian wild boars (*Sus scrofa*) exhibit severe clinical signs, whereas wild African swine species, such as warthogs (*Phacochoerus aethiopicus*) and bushpigs (*Potamochoerus porcus*), are highly resistant, remaining asymptomatic carriers and serving as reservoirs [3]. The virus is remarkably resilient in the environment, retaining infectivity for extended periods in matrices such as blood, meat, offal, soil, and fomites [4].

ASF was first reported in Kenya in 1921 and was initially endemic to sub-Saharan African countries [5]. In 2007, ASF was introduced to Georgia, spreading to neighboring countries, including Russia, Armenia, and Azerbaijan [6]. In 2014, the first occurrence of ASF was reported in the European Union, and many European countries have been affected [7]. In August 2018, the disease reached eastern China [8] and has continued to spread across Asia, with outbreaks confirmed in 20 countries as of December 31, 2024: China, Mongolia, Vietnam, Cambodia, North Korea, Laos, Myanmar, the Philippines, South Korea, Timor-Leste, Indonesia, Papua New Guinea, India, Malaysia, Bhutan, Thailand, Nepal, Singapore, Bangladesh, and Sri Lanka [9]. Papua New Guinea remains the only region in the Pacific to have experienced ASF outbreaks [10].

The epidemiology of ASF is described by four distinct cycles: sylvatic, tick-pig, domestic, and wild boar-habitat [7]. The domestic cycle accounts for most ASF outbreaks worldwide, where the virus spreads between domestic pigs or via pig-derived products without involving natural reservoirs [11]. In the wild boar-habitat cycle, ASFV circulates between wild boars and their habitat [12]. This cycle is particularly important in European countries and South Korea, where wild boars play a key role in virus transmission [13]. Additionally, contact between infected wild boars and domestic pigs is considered a major factor in the spread of ASFV in various regions, including Eastern Europe, the Caucasus, and the Russian Federation, where small backyard pig farms with inadequate biosecurity are common [14].

In Asia, the domestic cycle plays a major role in the spread of ASF [15]. Domestic pigs are abundant in the region, with China housing over 400 million pigs in 2024, the largest population globally [16]. The spread of the disease is driven by poor biosecurity, swill feeding practices, transportation of infected-pigs and pork products, and sociocultural practices [15, 17, 18]. Wild boar cases have been relatively uncommon in most Asian countries, with notable exceptions in South Korea and Malaysia, despite the predicted high density and wide distribution of wild boars [19]. Due to limited active surveillance in many Asian countries, infections in wild boars are likely underreported [15, 20, 21]. The combination of high wild boar density, extensive habitats, and low biosecurity on farms increases the risk of contact between wild boars and domestic pigs. This suggests that ASF outbreaks in wild boars pose a significant threat of spillover transmission to domestic pigs, further complicating disease control efforts [20, 22]. Therefore, both wild boars and domestic pigs should be considered important risk factors in the spread of ASF in Asia.

Geographic information system (GIS)-based multicriteria decision analysis (MCDA) is a systematic approach that integrates geographic data with evaluation criteria to provide a comprehensive assessment for decision-making [23–25]. While MCDA is a widely adopted method in various fields due to its flexibility and applicability [26–29], the selection of the evaluating group is crucial, as the characteristics of the group can significantly influence the results [30]. In this study, we adopted a similar approach, simplified multicriteria approach (SMCA), as an initial-stage method by equally allocating weights to all variables, thereby avoiding potential bias in determining risk weights. We set two distinct objectives across a wide geographical scale covering most of the Asian continent using SMCA-based on an exploratory risk analysis approach: (1) to identify vulnerable areas (VAs) for ASFV infection in domestic pigs and wild boars, and (2) to highlight the domestic pig–wild boar interface. These high-resolution maps are intended to serve as valuable spatial tools to enhance ASF management and surveillance efforts and improve control strategies for other diseases in Asian countries.

## 2. Materials and Methods

To create VA maps predicting VAs for ASFV infection in domestic pigs and wild boars, as well as their potential interactions, the following steps were undertaken: (1) defining the study area, (2) identifying predictor variables contributing to ASFV infection in each host, (3) transforming and standardizing the geographic layers of the predictor variables, (4) performing the SMCA and generating the VA maps for ASFV infection in both species, (5) evaluating the influence of each variable on the overall risk and results, and (6) overlapping both VA maps to delineate the domestic pig–wild boar interface.

### 2.1. Study Area

Given the broad study area, the climate, vegetation, and topography are highly diverse. Asia has a wide range of vegetation types, including tundra, coniferous forests, temperate grasslands, monsoon forests, equatorial forests, deserts, and Mediterranean forests, as classified by the Köppen Climate Classification Map [31]. The highest mountains on Earth, such as the Tibetan Plateau and the Himalayas, span the continent [32]. Furthermore, certain regions have particularly dense domestic pig populations [33].

All Asian countries, except Singapore, were selected for analysis. Singapore was excluded due to the absence of domestic pig farming and the minimal relevance of ASF's impact on the country's pig industry [34]. Despite its geographical isolation, Papua New Guinea was included because of its close interactions with Asia. Additionally, islands without roads or rivers were excluded.

### 2.2. Predictor Variables

To predict VAs for ASFV infection in domestic pigs and wild boars, three and 11 variables, respectively, were selected based on a literature review and expert knowledge (Table 1). These variables were broadly categorized into the following elements: climate, vegetation, topography, habitat suitability for wild boars, livestock density, and human impact. For wild boars, potential underreported areas were also considered.

All raster data were standardized to have the same projection, extent, and spatial resolution (30 arc-sec, ~1 km) using ArcGIS v10.8.1 and R v4.2.2 (R Core Team, 2022) with the “raster” [45], “dplyr” [46], “reshape2” [47], “ggplot2” [48], and “factoextra” packages [49]. Pearson correlation analysis in R v4.2.2 [50] was performed to address collinearity, and the remaining variables were applied in the subsequent SMCA.

#### 2.2.1. Predictor Variables for the Domestic Pig VA Map

For domestic pigs, we included pig density, the quality of available habitat (QAH) distribution for wild boars (Level 1: “suitable areas for food or shelter”), and human footprint density (Table 1). Domestic pigs play a significant role in the spread of ASFV, and pig density distribution has been identified as the most important predictor of ASF outbreaks in previous studies [23, 51, 52]. To mitigate the effect of upper outliers on value scaling, we capped values above 7355 heads/km^2^ at 7355, representing 1.7% of the pixels.

The QAH map developed by Bosch et al. [36, 53] is a cartographic tool that quantifies wild boar habitat using seven categories (0, 0.1, 0.5, 1, 1.5, 1.75, and 2). Although the QAH represents a proxy for wild boar habitat suitability rather than direct population density, this layer was validated across Eurasia [36], and has been effectively implemented in Southeast and East Asia, including Japan and South Korea [20, 54–56]. As described previously, contact between infected wild boars and domestic pigs poses a significant risk for ASF spread, particularly in low-biosecurity settings. QAH Level 1, representing areas suitable for food or shelter, has been identified as a zone where contact between the two species is likely [53]. For this analysis, QAH Level 1 areas were used, excluding desert regions where pig farming is unlikely.

The human footprint variable was defined as the percentage of relative human influence in each terrestrial biome. Human activities, such as the illegal movement of ASFV-infected pigs or the transport of contaminated fomites or products, contribute to the spread of the disease [57]. Therefore, this variable serves as an indirect indicator of urbanization and human activity, potentially reflecting trade frequency. Cities with populations exceeding 600,000 were excluded from the analysis to minimize their disproportionate influence on the results.

#### 2.2.2. Predictor Variables for the Wild Boar VA Map

For the wild boars, several factors were selected as explanatory variables (Table 1): topographic (terrain ruggedness index (TRI), slope, wild boar occurrence index by altitude), environmental (distance from rivers, forest coverage), climatic (temperature, precipitation, moisture index), habitat (wild boar distribution index), and underreporting factors (distance from roads, inverse of human footprint).

Previous reports have suggested that wild boars prefer habitats at higher elevations and areas with a greater proportion of slopes [58]. Therefore, we included the TRI and slope variables. TRI is the mean of the absolute differences in elevation for grid points, indicating the overall jaggedness or flatness of the terrain in an area [24]. We modified the altitude layer to better reflect wild boar distribution. To achieve this, the presence of wild boars was retrieved from several databases described in previous work [39]. The corresponding altitude value from the layer was assigned to each wild boar presence, and a Weibull probability distribution was fitted. Altitude values equal to or lower than zero were considered positive (0.1) in the transformation. This transformed variable is referred to as “altitude Weibull.” This process was conducted in R v4.2.2 using the “raster” [45], “sf” [59], “fitdistrplus” [60], “dplyr” [46], and “ggplot2” [48] packages. Areas with temperatures lower than −7°C, considered too harsh for wild boar habitat, were also excluded from subsequent analyses [39, 61].

Since water and food resources are vital for sustaining life, wild boar habitats are typically located near these resources [43]. Therefore, environmental factors such as forest coverage and distance from rivers were included. The raster values for distance from rivers were inverted to ensure that higher values represented closer proximity to water sources. The wild boar distribution index was generated by multiplying the average normalized difference vegetation index (NDVI) with the QAH level for wild boars, in line with previous studies [43]. NDVI, a metric used to assess the quantity, quality, and growth of vegetation, is frequently used as a predictor in wild boar distribution model [53, 62].

Climatic conditions, including temperature, precipitation, and humidity, have been identified in some studies as factors influencing the distribution of wild boars and ASF cases among wild boars [57]. Therefore, the variables annual mean temperature (bio1), annual precipitation (bio12), and annual mean moisture index (bio28) were included in the model [42].

Wild boar carcasses are more likely to be discovered in areas accessible to humans, while surveillance tends to be insufficient in regions farther from roads and human settlements [63]. Therefore, distance from roads and the human footprint were included to account for surveillance bias. These values were inverted to highlight the natural areas favorable for wild boar habitats. Raster layers for the distances to rivers and roads were generated at the spatial resolution used in this study. The distance from each cell to the nearest road or river was calculated using the “Euclidean Distance” tool in ArcGIS 10.8.1 [64]. Subsequently, collinearity among the selected variables was examined.

### 2.3. SMCA for Creating Predicted VA Maps for ASFV Infection in Domestic Pigs and Wild Boars

SMCA was applied to estimate the VA for ASF transmission in domestic pigs and wild boars. After the variable selection, raster pixel values for each variable were scaled using the “Raster Calculator” tool in ArcGIS v10.8.1 [65]. The scaled values were then combined with the “Weighted Sum” tool in ArcGIS v10.8.1 to compute the ASFV infection risk index [66], assigning equal weights to all variables. The calculation was conducted based on the following formula:

  $$\begin{array}{c} R\left(x\right)=\sum_{i=1}^{n}W_{i}S_{i}\left(x\right)\left(n=3\text{ for domestic pigs},\;n=6\text{ for wild boars}\right), \end{array}$$

where *R*(*x*) is the composite ASF risk score at location *x*, *n* is the number of predictor variables, *W*_*i*_ is the weight for predictor variable *i* at location *x*, and *S*_*i*_(*x*) is the standardized value of predictor variable *i* at location *x*. In this analysis, the weight for each variable (*W*_*i*_)  was set as equal.

Final risk values were derived by first standardizing each predictor and summing the resulting values. The aggregated values were then scaled from 0 to 1000, and classified into three risk levels-low, medium, and high-using Jenks natural breaks to facilitate clearer visual interpretation [67].

### 2.4. Evaluation of the VA Maps

To evaluate the impact of each variable on the overall risk, sensitivity analysis was conducted by adjusting the weight of each variable across five levels (0, 0.5, 1, 1.5, 2) and examining the resulting changes in average risk. This analysis was performed using the R v4.2.2 packages “terra” and “ggplot2.” The validity of the generated maps was assessed following the methods described in previous studies [36, 62]. Data on ASF notifications were obtained from March 1, 2018 to December 31, 2024 through the EMPRES Global Animal Disease Information System (EMPRES-i) of the Food and Agriculture Organization (FAO) of the United Nations [68]. Geographical coordinates of ASF notifications were overlaid on the VA maps. To ensure the reliability of the evaluation, areas within a buffer zone of 0.045° (~5 km) radius around each ASF notification were analyzed [36, 62]. The normalized density of notifications within each risk category was calculated by averaging the density of ASF notifications, defined as the number of notifications per number of raster cells within the buffer. A chi-square goodness-of-fit test was applied to compare the observed number of ASF notifications with the expected random distribution of notifications within the buffer. Statistical analyses were performed using the “chisq.test()” function in R.

### 2.5. Integration of VA Maps for Domestic Pigs and Wild Boars

Overlaying the infection VA maps for domestic pigs and wild boars provides insight into potential contact zones between the two hosts. The VA maps were combined using the “Weighted Sum” tool in ArcGIS 10.8.1. Considering the variation in the number of ASF notifications for domestic pigs and wild boars across Asian countries, equal risk assumptions for both hosts may not be justified. Therefore, risk weights for each host were adjusted at five levels: 1:9, 3:7, 1:1, 7:3, and 9:1, to evaluate the differences in their relative impacts on overall risk.

## 3. Results

### 3.1. Creation of VA Maps by SMCA

Variables with a Pearson correlation coefficient greater than 0.7 were excluded from the final analysis. The remaining variables described below were used in the SMCA. In the domestic pig study, no collinearity was observed among the selected variables (Figure S1A). In the wild boar study, after assessing collinearity, the following explanatory variables were chosen: topographic factors (TRI, altitude Weibull), environmental factors (distance from rivers, forest coverage), habitat factors (wild boar distribution index), and underreporting factors (distance from roads, inverse of human footprint, Figure S1B, Table 1). The final risk values for both domestic pigs and wild boars were adjusted to a scale of 0–1000. Risk levels were classified as follows: low risk (0 to 223 for domestic pigs, 0–400 for wild boars), medium risk (224 to 564 for domestic pigs, 401–580 for wild boars), and high risk (565 to 1000 for domestic pigs, 581–1000 for wild boars).

**Figure 1:**
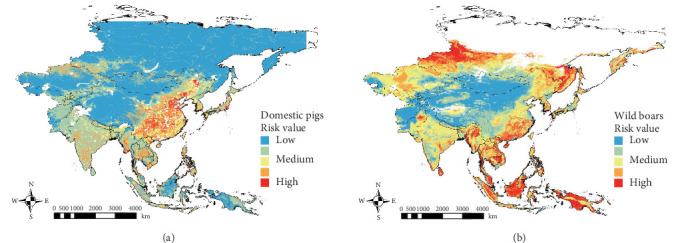
Predicted vulnerable areas (VA) for African swine fever (ASF) infection in Asia based on risk assessments for domestic pigs (a) and wild boars (b). The graded color maps represent the risk of ASFV infection from high (red) to low (blue) based on Jenks' natural breaks classification.

The spatial SMCA outputs indicated that areas highly vulnerable to ASFV infection in domestic pig populations were concentrated in eastern China, followed by other East Asian countries (South Korea and Japan), and Southeast Asia (Vietnam, Myanmar, and the Philippines). Conversely, the probability of ASFV infection in domestic pigs was low in Malaysia, Indonesia, Papua New Guinea, Mongolia, and Russia, except for border regions with China and Central Asia (Uzbekistan and Kazakhstan) (Figure 1a). By overlaying the VA map with ASF outbreak locations reported between March 2018 and December 2024, many outbreaks were observed in high- and medium- risk areas, including eastern China, the China–Russia border region, Vietnam, the Philippines, and South Korea (Figure S2A).

For the wild boar VA map, high- and relatively high-risk areas were identified in Russia, eastern China, Southeast Asia (Thailand, Vietnam, Cambodia, Myanmar, Malaysia, Indonesia, and Philippines), and Papua New Guinea (Figure 1b). The VA map overlaid with ASF cases in wild boars demonstrated a strong correspondence between reported ASF cases and predicted high-risk areas, particularly in Malaysia, South Korea, and the border region between China and Russia (Figure S2B). The factors contributing to the high risk of ASFV infection in wild boars varied by region. In Russia, distance from urban areas/roads and elevation were the primary determinants of high-risk zones, while in Southeast Asia, suitable habitat for wild boars and proximity to rivers were the key drivers of risk (Figure S3).

### 3.2. Validation of the VA Maps

**Figure 2:**
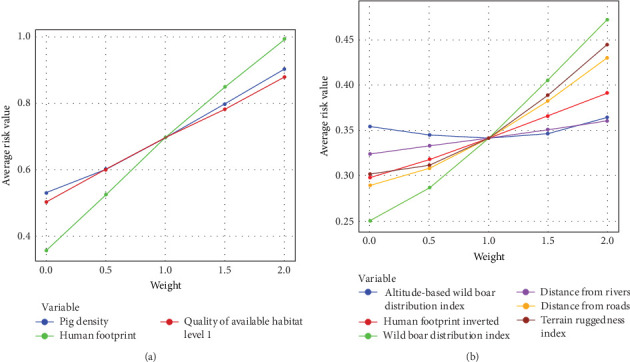
Results of the sensitivity analysis of the risk of ASFV infection in domestic pigs (a) and wild boars (b). Horizontal axis shows the weight change of the selected variables against the average risk value in the vertical axis.

The average risk of ASFV infection for the VA map resulting from the sensitivity analysis is shown in Figure 2, which reflects the sensitivity of each variable, with the more sensitive factors having a larger increase in the average risk value. The analysis revealed that human footprint was the most influential variable for ASFV infection in domestic pigs among three predictor variables, followed by pig density and QAH Level 1 for domestic pigs (Figure 2a). For wild boars, the wild boar distribution index was the most influential variable, followed by TRI, distance from roads, inverted human footprint, distance from rivers, and altitude Weibull (Figure 2b). To evaluate the predictive performance of the generated VA maps, 5066 ASF outbreaks in domestic pigs and 1773 wild boar ASF cases were analyzed. Statistically significant differences were observed between the number of reported cases (*n*_reported_) and the expected number of cases (*n*_expected cases_) in each area (*p* < 0.01, Table 2). In domestic pigs, the number of actual cases in high- and medium-risk areas was substantially greater than expected, whereas fewer cases than expected were observed in low-risk areas. Normalized density of notifications was highest in high-risk areas. For wild boars, the number of reported cases in high- and medium-risk areas closely matched the expected distribution, but the number of cases in low-risk areas was significantly lower than expected under a random distribution.

### 3.3. Potential Domestic Pig-Wild Boar Interface

**Figure 3:**
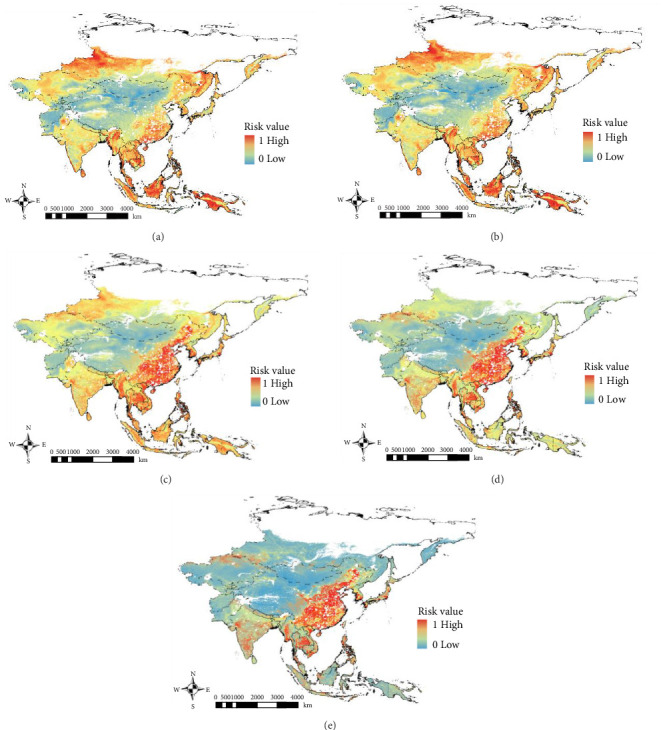
Potential interaction areas between domestic pigs and wild boars for ASF transmission, with varying risk weight assignments: (a) 1:9, (b) 3:7, (c) 1:1, (d) 7:3, and (e) 9:1. The risk value ranges from 0 (low risk) to 1 (high risk).

We identified regions where ASFV transmission between domestic pigs and wild boars is likely to occur. To assess the differences in their relative impact on overall risk, we assigned varying risk weights to the two hosts. The resulting VA map highlighted that high-risk areas for ASF spread between domestic pigs and wild boars were primarily concentrated in eastern and central China, the southwestern region of South Korea, and southern Japan, regardless of the weighting applied to the VA maps for domestic pigs and wild boars (Figure 3a–e). Additionally, relatively high-risk areas were observed in the Philippines and some regions in the Indochina peninsula. As the risk weight for wild boars increased, the risk in southern Russia and Southeast Asia rose (Figure 3a, b), whereas a higher risk weight for domestic pigs correlated with an increased risk in China (Figure 3d, e).

## 4. Discussion

In this study, we developed two maps estimating VA for ASFV infections in Asian domestic pigs and wild boars using spatial SMCA. We also predicted potential areas of ASF transmission between domestic pigs and wild boars across Asia. This study is the first to quantify the risk of ASFV infection across the broad Asian region based on environmental and anthropogenic factors, rather than solely on the geographic coordinates of reported ASF cases. This approach enables the prediction of ASF risk areas, including potentially underreported regions where ASF outbreaks have not been detected. It can thus improve the surveillance system, including early detection of the disease. Effective disease control requires addressing the issue beyond national borders and tackling it at the level of the broader Asian economic region as a whole, and the VA maps will be helpful in this regard.

Here, we positioned SMCA as an initial-stage analysis to identify ASF risk areas across Asia, and equal weights were assigned to each variable. This approach allows risk estimation without introducing preconceived notions about the importance of each variable. While MCDA is a powerful tool that determines risk weighting based on expert opinions, it has been noted that the importance assigned to variables may heavily depend on the selected experts' responses, potentially leading to bias [30].

In this study, we conducted sensitivity analyses to simulate the extent to which each predictor influenced the model output. In addition, we employed a chi-square goodness-of-fit test to evaluate whether the reported outbreaks deviated from a random distribution relative to our model. Although this test does not provide measures of sensitivity or specificity, alternative metrics such as the Area Under the Receiver Operating Curve (AUC) ROC curve were not applicable due to potential biases in the reported case data.

In domestic pigs, the sensitivity analysis showed that the human footprint has the greatest impact on the risk of ASFV infection in domestic pigs (Figure 2a). Since the Asian region holds the world's largest population and has many countries with high population densities, human activity is expected to have a stronger impact on the spread of the disease. Because detailed, harmonized, and reliable datasets on variables that directly reflect ASFV infection risk in domestic pigs—such as farm typology (e.g., commercial vs. backyard systems), pig movement routes, and trade volumes—are not publicly available in Asia, we relied on indirect proxies to approximate human-associated transmission risks. To provide a more realistic representation of biosecurity risks and potential exposure pathways, the incorporation of such proxies will be essential.

High-risk areas for domestic pigs were mainly concentrated in eastern China (Figure 1a). This aligns with previous studies that identified ASF high-risk areas in China, attributing the high risk to the dense pig populations and large swine farming areas along the coastal regions [51, 52]. In Southeast Asia, high-risk areas were also identified in countries with significant pork industries, such as Vietnam and the Philippines, where many ASF notifications were reported within high-risk areas. Pig farms in eastern China and Southeast Asia are largely small-scale or backyard farms with limited biosecurity measures [66, 67, 69, 70]. These findings highlight the need to strengthen biosecurity in high-risk areas in these countries. It should be noted that the risk levels classified using Jenks natural breaks represent relative, rather than absolute, values. This method is intended to highlight areas with comparatively higher risk and to support prioritization, not to establish fixed thresholds; therefore, the results should be interpreted with appropriate caution.

The chi-square goodness-of-fit test for domestic pigs showed that normalized density of observed cases was the highest in high-risk areas, followed by medium- and low-risk areas, suggesting that the risk areas were appropriately estimated. However, in high- and medium-risk areas, the number of observed cases was remarkably higher than the expected number (Table 2). This could be due to the exclusion of suburban areas around large cities in our study. Since human footprint values in urban areas heavily influenced the SMCA, large cities were removed from the VA map for domestic pigs to minimize the influence of outliers. However, some pig farms and slaughterhouses are located in large cities and on highways [19, 55]. Therefore, these farms and slaughterhouses should be considered separately from our analysis. In contrast to the high- and medium-risk areas, the expected number of cases in the low-risk areas exceeded the observed cases. This is probably due to underreporting of cases by farmers, which is a common concern for many Asian countries [15].

For wild boars, the sensitivity analysis for wild boars revealed that the wild boar distribution index, TRI, and distance from roads were the factors that strongly influenced ASFV infection risk (Figure 2b). Considering the wide distribution of wild boar habitat in Asia, the results obtained are reasonable, since the presence of wild boar is strongly associated with ASFV infection [71]. TRI and distance from roads are associated with underreporting of wild boars. Asia has many areas with rugged terrain and areas that are inaccessible to humans, making surveillance of ASFV infection in wild boars difficult. In these areas, ASFV could circulate among wild boars unnoticed, which may play a role in spreading ASFV infection. Therefore, it is important to be paid attention in high-risk areas, even in areas where there are no reported ASF outbreaks in wild boars.

Most areas, except deserts and high mountain regions of Central Asia, were classified as medium to high-risk for wild boars (Figure 1b). The high-risk areas for wild boars were more widespread than those for domestic pigs, particularly extending into southern Russia and Southeast Asia. Since 2019, intensive ASF outbreaks have been reported in the Russian Far East region [71, 72], highlighting the significant migration patterns of wild boars in this area [73, 74]. Central Russia is less critical as a high-risk area compared to regions near Europe and the Russian Far East [73]. However, the potential for ASF spread in central Russia cannot be completely dismissed, as wild boars also inhabit northern Eurasia [75]. In Southeast Asia, while ASF outbreaks in wild boars have been limited, they have been reported several times [22]. The highest ASFV infection risk for wild boars in Asia is concentrated in Southeast Asia, where a variety of wild boar species are widespread [76]. These findings suggest the importance of surveillance in the entire southern Russia and Asian region.

The chi-square test of goodness of fit for wild boars showed that the normalized density in high-risk areas was comparable to that in medium-risk areas (Table 2). The number of ASF cases in wild boars across Asia may be higher than reported, given that many developing countries have not established sufficient surveillance systems [10, 20].

High-risk areas for potential ASFV transmission between domestic pigs and wild boars were identified in eastern China, South Korea, Japan, the Philippines, and some isolated regions in the Indochina peninsula (Figure 3). The fact that 99% of pig farms in China are small-scale operations, combined with a large and widely distributed wild boar population, suggests a significant risk for ASFV transmission from wild boars to domestic pigs [16, 70, 76]. In South Korea, the spillover of ASFV from wild boars to domestic pigs presents a major threat to the country's pig industry [13]. In Vietnam and the Philippines, ASF outbreaks in domestic pigs have spread across the country, and the endemic species of wild boars are at high risk of ASFV infection [76]. In Japan, while ASF has not yet been reported, classical swine fever, which shares epidemiological characteristics with ASF, is spreading primarily among wild boars and has had a significant impact on the spread of infection in pig farms [54]. From this perspective, our map can help identify high-risk areas for ASFV transmission between both species, not only in ASF-endemic countries but also in regions where ASF is not currently present. This can facilitate the implementation of rapid control measures for both domestic pigs and wild boars in the event of an ASF outbreak.

There are several limitations in this analysis. First, due to constraints in data availability across countries, the SMCA was applied to a broad study region; however, the actual importance of each variable is unlikely to be uniform. To partially address this limitation, we conducted a sensitivity analysis by varying the weights assigned to each variable. This analysis allowed us to identify the factors that exert the greatest influence on risk estimation. The SMCA approach serves an exploratory role by generating an unbiased baseline. Nevertheless, for country-level ASF risk assessments, it will be essential to incorporate local expertise, together with region-specific data and evidence from the literature. Such integration will help establish more context-appropriate weightings, reduce potential bias, and enhance the applicability of MCDA. Second, because data reflecting seasonal trends or other time-varying factors were not available, temporal elements were excluded from this analysis. However, as seasonal variation in ASF outbreaks has been reported in several regions of Asia [15, 77], incorporating temporal dynamics should be considered in future research to enhance the predictive capacity and accuracy of risk models. Third, although we selected the most recent datasets available at the time of initiating this study, there is time variation when the different predictor variables, as well as the ASF outbreak data, were recorded. Therefore, we cannot rule out the possibility that this temporal variability may have influenced our results. Future studies could improve predictive accuracy by employing approaches that account for such temporal mismatches.

In summary, we assessed the risk areas for ASFV infection in both domestic pigs and wild boars across Asia, including the potential interaction between these two hosts. Through sensitivity analysis, we identified key risk factors that significantly contribute to ASFV infection risk, providing valuable insights into disease dynamics. A standardized tool to compare ASFV infection risks by country and across different hosts would be valuable for facilitating cross-border cooperation and enhancing risk management at the national level. To eradicate the disease, it is crucial to accurately assess the epidemic situation and evaluate the associated risks. Our tool can also be adapted for monitoring other transboundary swine diseases in Asia.

## Figures and Tables

**Table 1:** Predictor variables used in the multicriteria decision analysis for predicting African swine fever infection risk.

Category	Variable description	Selected variables after the collinearity check	Year	Data origin
**Domestic pig**				

Livestock	Pig density	Yes	2015	[35]
Wild boar	Quality of available habitat (QAH) level 1^a^	Yes	2017	[36]
Anthropogenic	Human footprint	Yes	2004	[37]

**Wild boar**				

Topography	Terrain Ruggedness Index (TRI)^a^	Yes	2018	[24]
	Slope	No	2008	[38]
	Altitude-based wild boar distribution index (altitude Weibull)^a^	Yes	2020	[39]
Environment	Distance from rivers^b^	Yes	2017	[40]
	Forest coverage	No	2004–2022	[41]
Climate	Annual mean moisture index (bio28)	No	2017	[42]
	Annual precipitation (bio12)	No	2017	[42]
	Annual mean temperature (bio1)	No	2017	[42]
Habitat	Wild boar distribution index (NDVI × QAH)^a^	Yes	2000–2021	[36, 41, 43]
Underreporting	Distance from roads^b^	Yes	1980–2010	[44]
	Human Footprint^b^	Yes	2004	[37]

^a^modified.

^b^inverted.

**Table 2:** Results of the chi-square test of goodness of fit results for African swine fever notifications.

Risk level	*n*_reported_		Normalized density		*n*_expected cases_		Residual	
	Domestic	Wild	Domestic	Wild	Domestic	Wild	Domestic	Wild
Low	1301	25	11489.5	16926.2	2090.2	66.3	−17.3	−5.1
Medium	2469	733	21087.2	47921.0	2161.3	686.7	6.6	1.8
High	1296	1015	29373.1	44670.4	814.5	1020.0	16.9	−0.2
Total	5066	1773						

*Note:* Expected cases were estimated by randomly distributing ASF cases across the occupied cells within the study area. For domestric pigs, *χ*^2^ = 626.43, df = 2, *p* < 0.01. For wild boars, *χ*^2^ = 28.873, df = 2, *p* < 0.01. Domestic: domestic pigs; Wild: wild boars.

## Data Availability

All variables used in this study are publicly available, with their sources detailed in the “Materials and Methods” section. The datasets generated during the current study are available via the following request form:https://docs.google.com/forms/d/e/1FAIpQLSdXaClR0Nx-8-awupI9h37GzBzlAmabUyFlFv2nJTsoK66J_A/viewform?usp=sharing. All other relevant data are available from the corresponding author upon reasonable request.

## Supporting Information

Supporting Information Figure S1: Pearson correlation matrix of variables used in the risk assessment for domestic pigs (A) and wild boars (B). Figure S2. Predicted vulnerable areas (VA) overlaid with ASF notification points for domestic pigs (A) and wild boars (B), respectively. Figure S3. Maps of each predictor variable rescaled to a 0–1 range for domestic pigs (A) and wild boars (B), respectively.
