# Supplementary material for: Risk Mapping of African Swine Fever in Domestic Pigs and Wild Boars to Enhance Management and Surveillance in Asia
Source: Transbound Emerg Dis. 2025 Nov 21;2025:8850856. doi: 10.1155/tbed/8850856 (PMC12662683; doi:10.1155/tbed/8850856)
Supplement: Supporting Information — Figure S1: Pearson correlation matrix of variables used in the risk assessment for domestic pigs (A) and wild boars (B). Figure S2. Predicted vulnerable areas (VA) overlaid with ASF notification points for domestic pigs (A) and wild boars (B), respectively. Figure S3. Maps of each predictor variable rescaled to a 0–1 range for domestic pigs (A) and wild boars (B), respectively. [file 8850856.f1.docx]

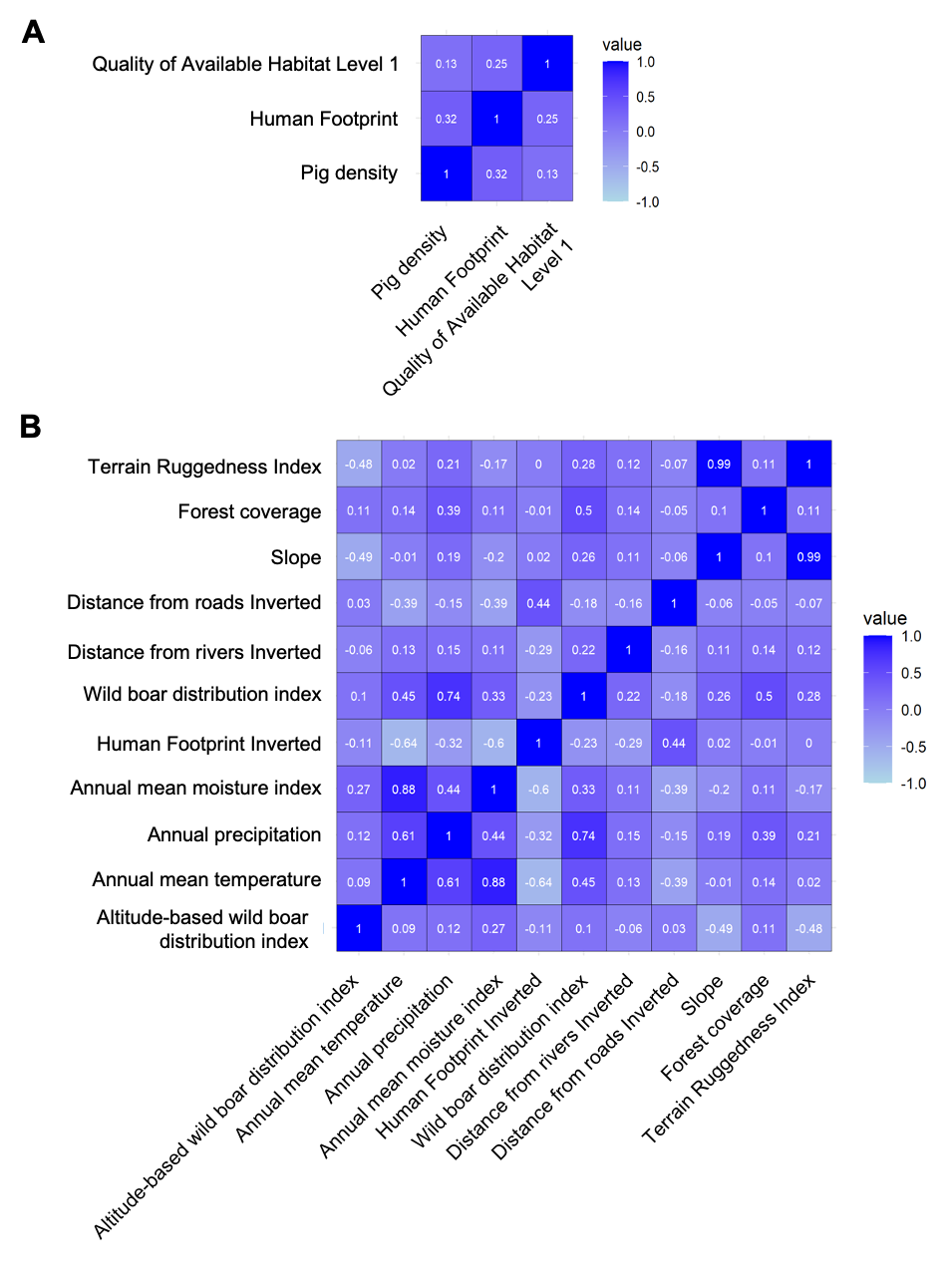


**Supplementary Figure 1.** Pearson correlation matrix of variables used in the risk assessment for domestic pigs (A) and wild boars (B).


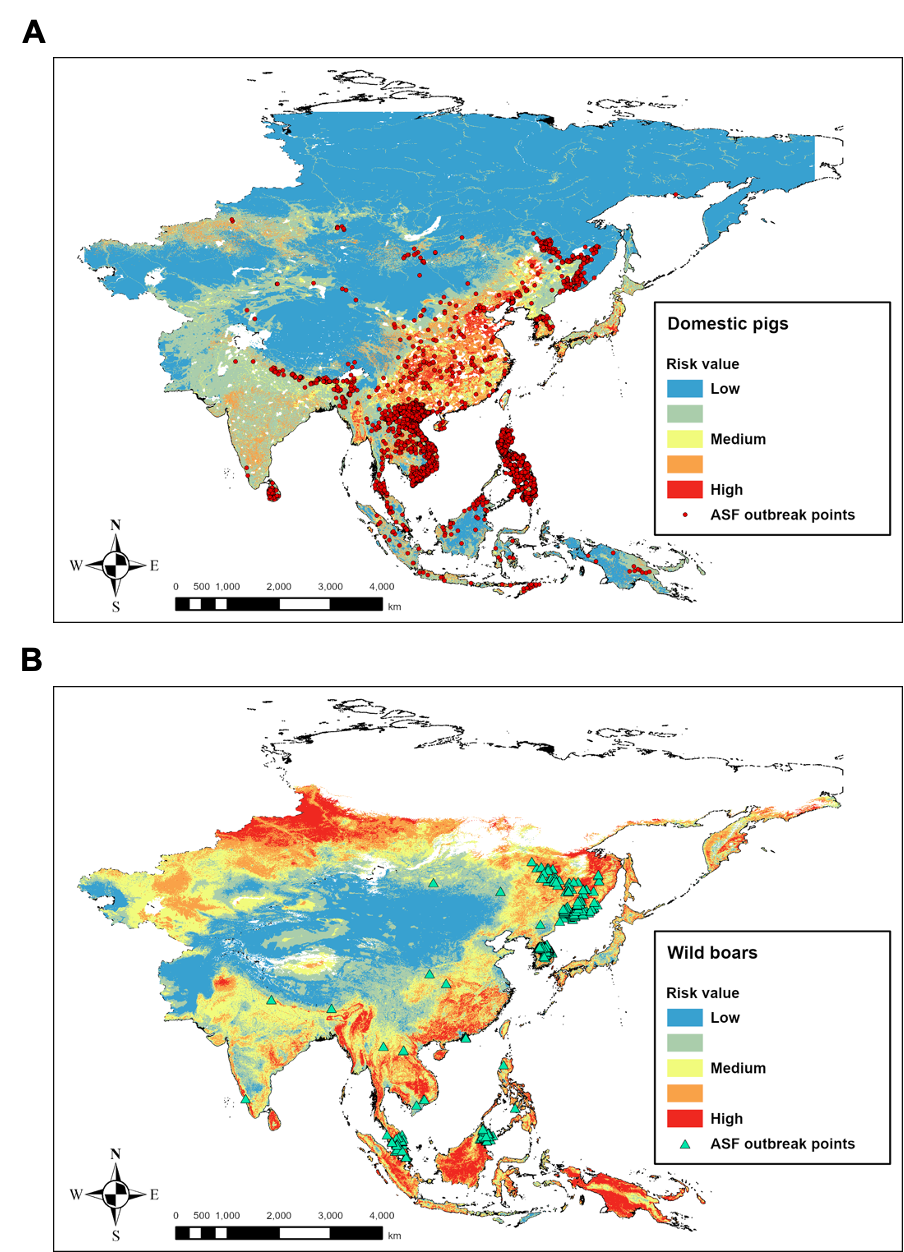


**Supplementary Figure 2.** Predicted vulnerable areas (VA) overlaid with ASF notification points for domestic pigs (A) and wild boars (B), respectively.


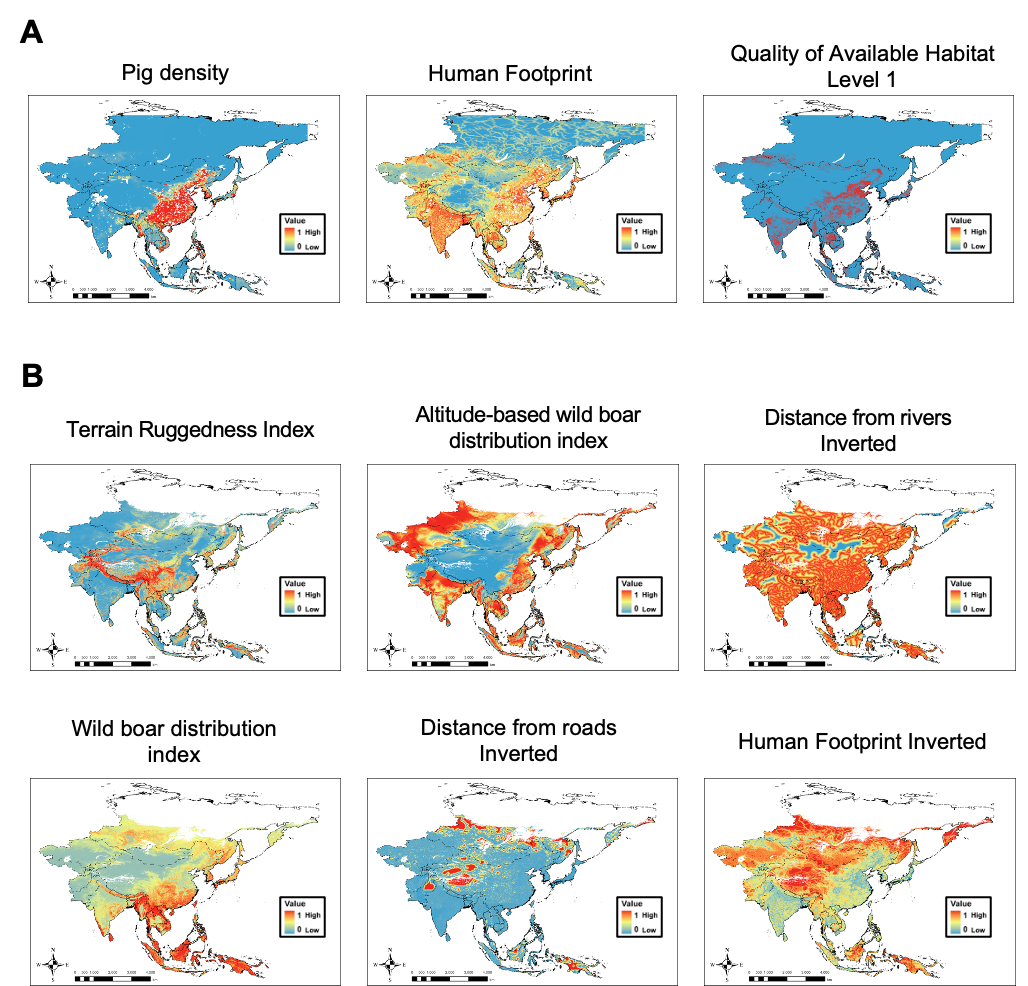


**Supplementary Figure 3.** Maps of each predictor variable rescaled to a 0-1 range for domestic pigs (A) and wild boars (B), respectively.
